# Pharmacological Evaluation of *Prosopis cineraria* (L.) Druce in Gastrointestinal, Respiratory, and Vascular Disorders

**DOI:** 10.1155/2012/735653

**Published:** 2012-12-05

**Authors:** Khalid Hussain Janbaz, Sajjad Haider, Imran Imran, Muhammad Zia-Ul-Haq, Laura De Martino, Vincenzo De Feo

**Affiliations:** ^1^Faculty of Pharmacy, Bahauddin Zakariya University, Multan, Pakistan; ^2^Department of Pharmacognosy, Research Institute of Pharmaceutical Sciences, University of Karachi, Karachi 75270, Pakistan; ^3^Department of Pharmaceutical and Biomedical Science, University of Salerno, Via Ponte don Melillo, Fisciano, 84084 Salerno, Italy

## Abstract

In this paper, a crude methanolic extract from the stem bark of *Prosopis cineraria*, a plant native of Pakistan, was tested for its possible presence of spasmolytic, bronchodilator, and vasodilator activities in an attempt to validate some of its folkloric uses. Moreover, attempts were made to provide plausible explanations of the observed biological activities. The extract caused relaxation of the spontaneous as well as K^+^ (80 mM)-induced contractions at tissue bath concentrations of 3–10 mg/mL in isolated rabbit jejunum preparations, probably mediated through blockade of Ca^+2^ channels. This finding was further confirmed by the shifting of the Ca^+2^ concentration response curves to the rightward in a manner similar to verapamil used as a standard Ca^+2^ channel blocker. The extract also exhibited nonspecific relaxant effect on carbachol (1 **μ**M)- and K^+^ (80 mM)-induced contractions in isolated rabbit tracheal preparations. The same effect was recorded for phenylephrine (11 **μ**M) and K^+^ (80 mM)-induced contractions in isolated rabbit aortic preparations in a manner similar to verapamil. These observations confirm that observed bronchodilator and vasodilator activities were possibly mediated through blockade of Ca^+2^ channels. The above-mentioned observations validate the traditional use of the plant in the treatment of respiratory and gastrointestinal ailments.

## 1. Introduction

*Prosopis cineraria* (L.) Druce (Leguminosae) locally known as ghaf, jand, jandi, and khejri, is a multipurpose indigenous tree growing wild in dry and arid regions of Pakistan [1, 2]. It is used by native healers to manage multiple ailments including gastrointestinal, respiratory, and cardiovascular disorders. The stem bark has folkloric repute to possess anti-inflammatory, antirheumatic, tonic, and vermifuge properties and is used in the treatment of anxiety, asthma, bronchitis, dyspepsia, fever, dysentery, leprosy, piles, wandering of the mind, and tremors. Furthermore, it is claimed to have abortifacient and laxative properties [3]. The smoke of the burnt leaves is used to treat eye inflammations. Leaf paste is applied on boils and blisters, including mouth ulcers in livestock and leaf infusion on open sores on the skin [4]. Flowers are used as an antidiabetic agent and to prevent abortion. The plant material is one of the herbal remedies for snake bite and scorpion sting [5]. The wood ash may be used as source of potash and the ashes are rubbed over the skin to remove hair. The leaves are good fodder for camels, goats, and donkeys. The pods are used as a vegetable [6]. 

Phytochemical investigations on the leaves of the plant resulted in the presence of hydrocarbons and phenolic acid derivatives [7, 8]. The structural elucidation of seed galactomannan was also reported [9].

Recent studies showed the antitumor activity of the hydroalcoholic extract of the leaves and stem barks of *P. cineraria* against Ehrlich ascites carcinoma-induced in mice [10] and the analgesic activity of an ethanol extract of roots [11]. Different extracts of stem bark possessed a weak antibacterial activity [12]. Antihyperglycemic, antihyperlipidemic, and antioxidative potential of *Prosopis cineraria *bark has been recently demonstrated [13].

Due to some of the traditional uses of the plant, including asthma and dysentery, the present study was undertaken to validate these folkloric uses.

## 2. Materials and Methods

### 2.1. Plant Materials and Preparation of Crude Extract

Fresh barks of *Prosopis cineraria *were collected from residential areas of Bahauddin Zakariya University, Multan, Pakistan in April 2010 and identified by Ms. Saima Shehzadi at the Institute of Pure and Applied Biology, Bahauddin Zakariya University, Multan. A voucher specimen deposited in herbarium of the same Institute (P.FL. 383-2). The plant material was shade dried for two weeks and was grinded in a herbal grinder. The powdered material (1 kg) was soaked in 70% aqueous methanolic for 7 days in glass bottles with occasional shaking. The soaked material was passed through double layered muslin cloth to remove vegetative debris. The marks were pressed and the obtained fluid was subsequently filtered through filter paper. Filtrate was evaporated on a rotary evaporator under reduced pressure at 37°C to a thick, semisolid mass. The crude extract was subjected to freeze drying to remove moisture; the approximate yield was 3.85%. The dried extract was transferred to air tight amber glass bottle and stored at −40°C in a refrigerator. Fresh dilutions of the crude extract were made on the day of experiment. 

### 2.2. Chemicals

Acetylcholine perchlorate, dicyclomine, atropine sulphate, carbachol, verapamil hydrochloride, phenylephrine, potassium chloride, magnesium chloride, and EDTA were purchased from Sigma Chemicals Co., St. Louis, MO, USA. Calcium chloride, glucose, magnesium sulphate, potassium dihydrogen phosphate, sodium bicarbonate, sodium dihydrogen phosphate, and sodium chloride were procured from Merck, Darmstadt, Germany, whereas sodium chloride, sodium hydroxide, and ammonium hydroxide were obtained from BDH Laboratory Supplies, Poole, UK. All the chemicals used were of highest purity reagent analytical grade. Stock solutions of the chemicals were made fresh in distilled water on the day of experiment.

### 2.3. Animals and Housing Conditions

Locally available (♂ & ♀) rabbits (1–1.8 kg) were purchased from market and maintained at 23–25°C at the animal house facility at Faculty of Pharmacy, Bahauddin Zakariya University, Multan. The animals were provided with fresh green fodder and tap water *ad libitum*. The animals were deprived of food 24 h prior to the experiment but had free access to water. The rabbits were sacrificed following a blow on back of the head and dissected to remove jejunum, trachea, and aorta for *in vitro* isolated tissue experiments. Experiments were performed in compliance with the rulings of the Institute of Laboratory Animals Resource, Commission on Life Sciences, National Research Council [14] and approved by the Ethical Committee of the Bahauddin Zakariya University, Multan.

### 2.4. Antispasmodic Activity on Isolated Rabbit Jejunum Preparations

The antispasmodic activity of plant material was studied using isolated rabbit jejunum preparations as described by Gilani and coworkers [15, 16]. Rabbit jejunum was cut into segments 2 cm in length and freed from adhering mesenteries. Each segment of tissue was mounted between two stainless steel hooks in a l0 mL tissue bath containing normal Tyrode's solution (pH 7.4), maintained at 37°C and aerated with carbogen (a mixture of 5% CO_2_ and 95% O_2_). A preload of 1 g was applied and spontaneous contractions were recorded isotonically through a Power lab Data Acquisition System (AD Instruments, Sydney, Australia). The tissues were allowed to be equilibrated for a period of 30 min prior to the addition of any drug and the tissue was washed repeatedly with fresh fluid at 10 min intervals. After equilibration, the spasmolytic action of the test material was assessed following addition of such material to isolated tissue bath in a cumulative fashion. The test material-induced relaxant effect was measured as percent decrease in amplitude of spontaneous contractions of isolated rabbit jejunum preparation recorded immediately prior to the addition of test material.

### 2.5. Determination of Ca^+2^ Channel Blocking Activity

The mechanism of the possible relaxant activity on the spontaneous contractions of test material was determined by the possible effect on high K^+^ (80 mM)-induced contractions in isolated rabbit jejunum preparations as described by Farre and coworkers [17]. The isolated rabbit jejunum preparations on exposure to K^+^ (80 mM) in the tissue bath exhibited a sustained contraction and the test material was added subsequently to the tissue bath, in a cumulative fashion to obtain concentration-dependent relaxant responses [18]. The extent of relaxation of precontracted isolated rabbit jejunum preparation was expressed as percent of the contractile responses exerted by K^+^ (80 mM). The K^+^ (80 mM)-induced smooth muscle contractions have been reported to be mediated through the influx of Ca^+2^ from extracellular fluid and agents capable to relax such contractions are speculated to be acting through blockade of Ca^+2^ channels [19]. The calcium channel blocking activity on the part of test material was confirmed further by the methods described previously by Gilani and coworkers [15, 16]. The isolated rabbit jejunum preparation was allowed to stabilize in normal Tyrode's solution, which was subsequently replaced with normal K^+^ and Ca^+2^ free Tyrode's solution containing EDTA (0.1 mM) for 45 min to ensure removal of Ca^+2^ from the tissue. The solution was replaced further by K^+^ rich and Ca^+2^ free Tyrode's solution of the following composition (mM): KCl (50), NaCl (91.04), MgCl_2_ (1.05), NaHCO_3_ (11.90), NaH_2_PO_4_ (0.42), glucose (5.55), and EDTA (0.1). After 30 min incubation the Ca^+2^ was added to the tissue bath in a cumulative manner to obtain control over Ca^+2^ concentration-response curves (CRCs). The observed stepwise increase in contractile activity of the isolated tissue preparation indicated that contractile activity is dependent on K^+^-induced influx of extracellular Ca^+2^. Subsequent to recording the super imposable control CRCs for Ca^+2^, the tissue was washed and equilibrated in the presence of the test material for 60 min. The CRCs for Ca^+2^ were recorded again and compared to the control curves. The CRCs for Ca^+2^ were also recorded in the presence of various concentrations of test material and Ca^+2^ channel blocking activity of test material was found to be confirmed subsequent to concentration-dependent shifting of the CRCs for Ca^+2^ towards right in Ca^+2^-free medium [19].

### 2.6. Bronchodilator Activity on Isolated Rabbit Tracheal Preparations

The rabbit tracheal tube was cut into rings of about 3-4 mm in width, each containing about two cartilages. Each ring was opened by longitudinal cut on ventral side opposite to the smooth muscle layer, forming a tracheal strip with a central part of smooth muscle sandwiched between cartilaginous portions on the edges. The preparation was suspended in a 20 mL tissue bath containing Krebs physiological salt solution at 37°C and aerated continuously with carbogen. A tension of 1 g was applied to each of tracheal strip and was kept constant throughout the experiment. The isolated rabbit tracheal preparation was equilibrated for 45 min prior to recording of isometric contractions via force displacement transducers connected to a Powerlab Data Acquisition System (AD Instruments, Sydney, Australia) and displayed on computer running Lab Chart software (version 6). The relaxant effect of the test material was assessed on carbachol (1 *μ*M)- and K^+^ (80 mM)-induced contractions in isolated rabbit tracheal preparation as cumulative addition of the test material to the isolated tissue bath may relax the isolated rabbit tracheal preparation.

### 2.7. Vasodilator Activity on Isolated Rabbit Aorta Preparations

The thoracic aorta was cut into rings of about 2-3 mm width and each ring was mounted in a tissue bath containing Krebs solution maintained at 37°C and aerated continuously with carbogen. A preload of 2 g was applied to each preparation and tissue was allowed to be equilibrated for 1 hr. The vasoconstrictive effect of the test material was assessed on cumulative addition of the test material to tissue organ bath containing tissue, whereas vasorelaxant was noted on cumulative application to isolated tissue bath containing tissue preparation already contracted by phenylephrine (1 *μ*M) as well as K^+^ (80 mM). The changes in isometric tension of the aortic rings were recorded via force-displacement transducer (Model FORT100, WPI, USA) attached to a Powerlab Data Acquisition System (AD Instruments, Sydney, Australia) and displayed on computer running Lab Chart software (version 6).

### 2.8. Statistical Analysis

The data were expressed as mean ± standard error of the mean (SEM) and the median effective concentrations (EC_50_ values) are given with 95% confidence intervals (CI). The statistical parameters were compared with *χ* ^2^-test and *P* < 0.05 was considered as significantly different.

## 3. Results

### 3.1. Effect on Isolated Rabbit Jejunum Preparations

**Figure 1:**
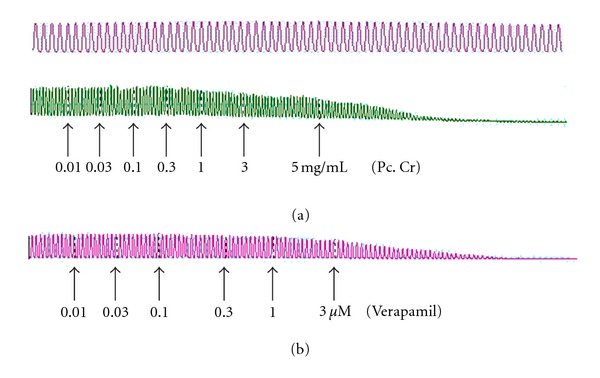
Effect of: (a) a methanolic extract of *Prosopis cineraria *(Pc. Cr) and (b) verapamil on spontaneously contracting isolated rabbit jejunum preparations. Plant extract was added in cumulative manner and the values listed were the final tissue bath concentrations.

**Figure 2:**
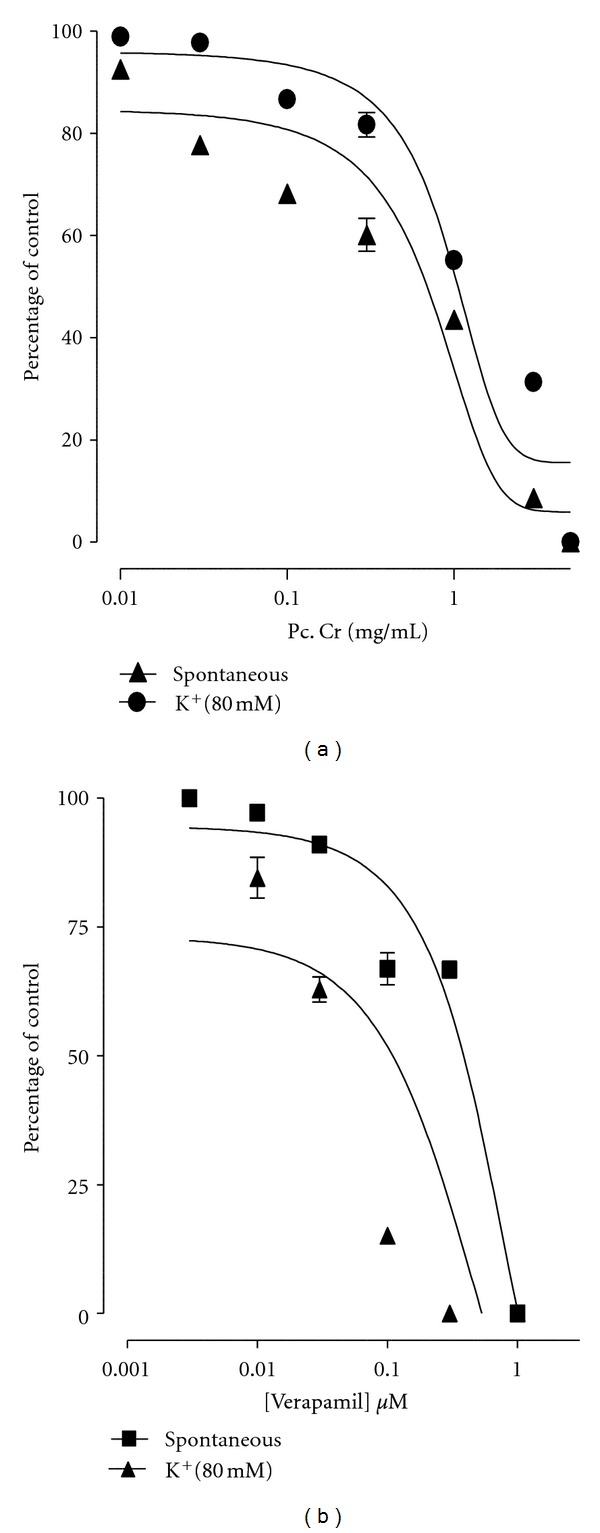
Concentration-dependent-relaxant effect of (a) a methanolic extract of *Prosopis cineraria *(Pc. Cr) and (b) verapamil on spontaneous and high K^+^ (80 mM)-induced contractions in isolated rabbit jejunum preparations (values are the mean ± SEM, *n* = 3–5).

The methanolic extract of *P. cineraria* caused relaxation of the spontaneous contractions in isolated rabbit jejunum preparations at concentration range of 0.01–5.0 mg/mL, with an EC_50_ value of 0.835 mg/mL (95% CI 0.0925–3.102, *n* = 3–5) (Figure 1). The extract also caused relaxation of K^+^ (80 mM)-induced contractions at concentration range of 0.3–5.0, mg/mL with an EC_50_ value of 2.015 mg/mL (95% CI 0.275–3.75, *n* = 3–5) (Figure 2(a)). The reference drug verapamil also caused relaxation of the spontaneous as well as K^+^ (80 mM)-induced contraction in isolated rabbit jejunum preparations with respective EC_50_ value of 0.767 *μ*M (95% CI 0.582–1.00; *n* = 5) and 0.389 *μ*M (95% CI 0.0055–0.743; *n* = 5) (Figure 2(b)).

### 3.2. Effect on Ca^2+^ Concentration-Response Curves

**Figure 3:**
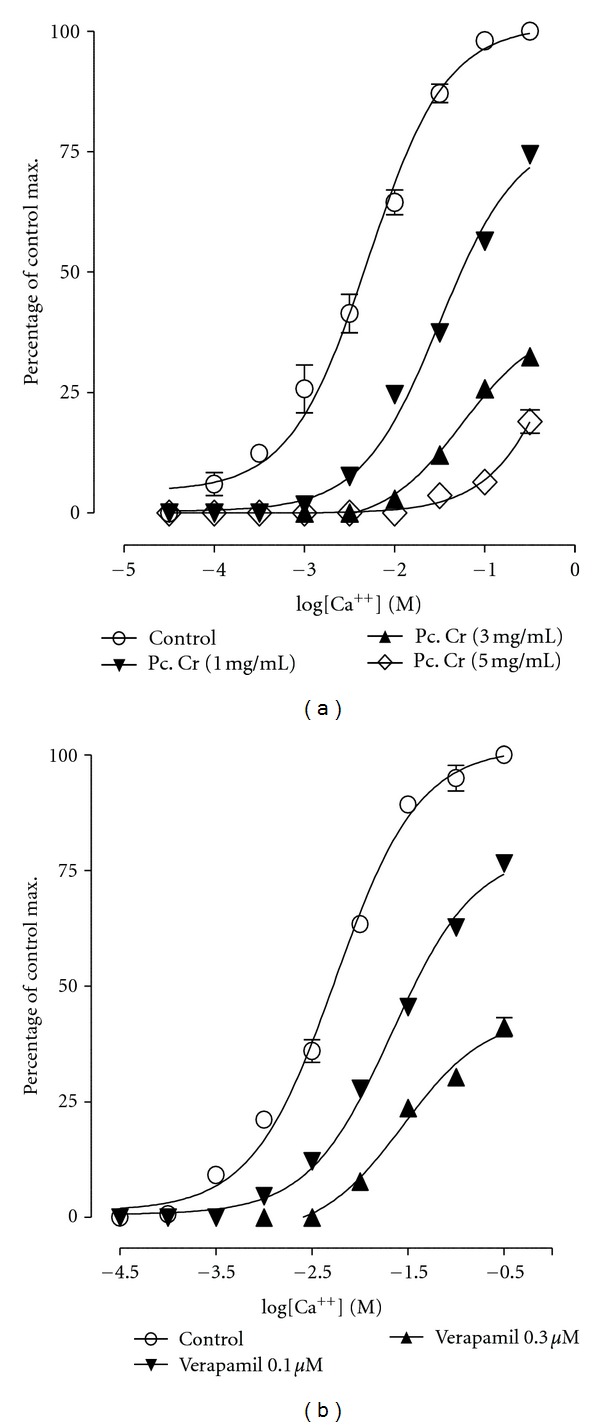
Effect of (a) a methanolic extract of *Prosopis cineraria* (Pc. Cr) and (b) verapamil on concentration response curves of Ca^+2^ in isolated rabbit jejunum preparations (values are the mean ± SEM, *n* = 3–5). Control = Ca^+2^.

The effect of the methanolic extract was assessed on the concentration-response curves (CRCs) for Ca^+2^. The spontaneous contractions in isolated rabbit jejunum preparations were observed to be suppressed following the depletion of Ca^2+^ from physiological salt solution, which was found to be revived on stepwise addition of Ca^2+^. Maximal contractile activity (100%) was recorded on restoration of Ca^2+^ concentration in tissue bath to its optimal level (6.4 mM). The extract at multiple tissue bath concentrations (1.0–5.0 mg/mL) shifted the CRCs for Ca^2+^ towards right (Figure 3(a)) in a manner comparable to the shifting caused by the presence of verapamil (0.1–0.3 *μ*M) (Figure 3(b)).

### 3.3. Effect on Isolated Rabbit Tracheal Preparations

**Figure 4:**
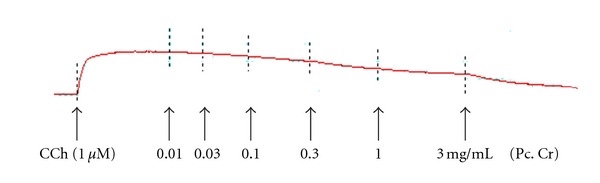
Concentration-dependent relaxant-effect of a methanolic extract of *Prosopis cineraria* (Pc. Cr) on carbachol (CCh, 1 *μ*M)-induced contractions in isolated rabbit tracheal preparation.

**Figure 5:**
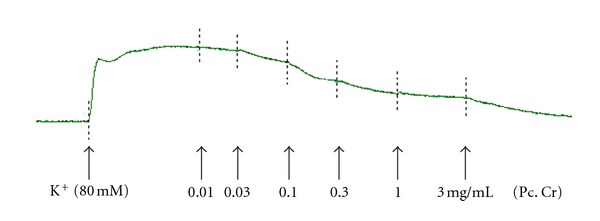
Effect of methanolic extract of *Prosopis cineraria *(Pc. Cr) on K^+^ (80 mM)-induced contractions in isolated rabbit tracheal preparation.

**Figure 6:**
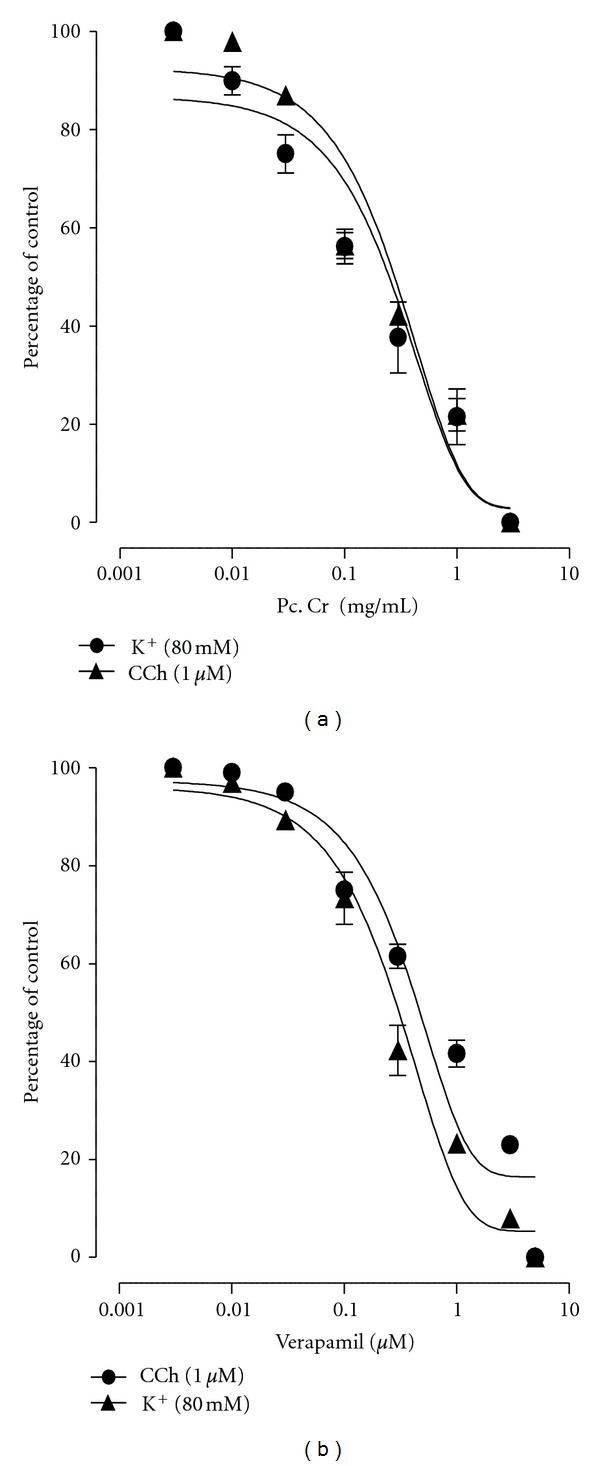
Concentration dependent inhibitory effect of: (a) a methanolic extract of *Prosopis cineraria *(Pc. Cr) and (b) verapamil on carbachol (1 *μ*M)- and K^+^ (80 mM)-induced contractions in isolated rabbit tracheal preparations (values are the mean ± SEM, *n* = 5).

The methanolic extract caused a complete relaxation of carbachol (1 *μ*M)- and high K^+^ (80 mM)-induced contractions in isolated rabbit tracheal preparation in concentration-dependent manner, with respective EC_50_ values of 0.568 mg/mL (95% CI 0.0244–2.80; *n* = 5) and 0.586 mg/mL (95% CI 0.010–2.80; *n* = 5) (Figures 4, 5, and 6(a)). Similarly, verapamil also caused the relaxation of carbachol (1 *μ*M)- and K^+^ (80 mM)-induced contractions, with EC_50_ values of 0.813 *μ*M (95% CI 0.0225–3.25; *n* = 5) and 0.550 *μ*M (95% CI 0.059–3.25; *n* = 5), respectively (Figure 6(b)).

### 3.4. Effect on Isolated Rabbit Aorta Rings Preparations

**Figure 7:**
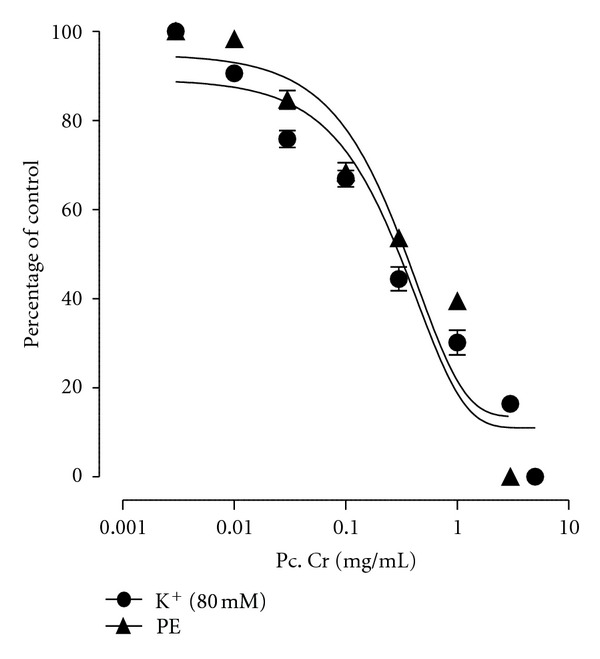
Effect of a methanolic extract (Pc. Cr) of *Prosopis cineraria *on phenylephrine (PE) (1 *μ*M)- and K^+^ (80 mM)-induced contraction in isolated rabbit aorta (values are the mean ± SEM, *n* = 5).

The methanolic extract on cumulative addition to isolated tissue baths caused a concentration-dependent relaxation of phenylephrine (1 *μ*M)- and K^+^ (80 mM)-induced contractions in isolated rabbit aorta rings, with respective EC_50_ values of 0.513 mg/mL (95% CI 0.0078–2.225; *n* = 5) and 0.525 mg/mL (95% CI 0.175–2.225; *n* = 5) (Figure 7).

## 4. Discussion

*Prosopis cineraria* has traditionally been used for the management of diarrhea/dysentery [5], and it was subjected to pharmacological screening on isolated rabbit jejunum to validate this folkloric use. The methanolic extract caused relaxation of the spontaneous contractions in isolated rabbit jejunum preparations in a concentration-dependent manner. The extract was tested on K^+^ (80 mM)-induced contractions in isolated rabbit jejunum preparations to explore the possible mechanism of the observed relaxant activity on spontaneous contractions, because smooth muscles when exposed to K^+^ (80 mM) are known to exhibit contractile response due to influx of extracellular Ca^+2^ through opening of the voltage dependent slow Ca^+2^ channels [19, 20]. The extract exerted a concentration-dependent relaxant effect on K^+^ (80 mM)-induced contractions, which may be due to blockade of Ca^+2^ channels. The proposed blockade of Ca^+2^ influx was confirmed further observing the rightward shifting of the concentration-response curves (CRCs) for Ca^+2^ in the Ca^+2^ free and K^+^ rich medium, similar to those provoked by verapamil [21]. Interestingly, standard Ca^+2^ channel blocking agents are known to exhibit spasmolytic and antidiarrhoeal activities [22]. 

*Prosopis cineraria* has been used traditionally and also the treatment of respiratory diseases like asthma, cough, and bronchitis. Hence, its methanolic extract was tested for its possible bronchodilator activity on carbachol (1 *μ*M)- and K^+^ (80 mM)-induced contractions in isolated rabbit tracheal preparations. The extract exerted a concentration-dependent relaxant effect on both of carbachol (1 *μ*M)- and K^+^ (80 mM)-induced contractions. The observed bronchodilator activity may possibly be mediated through blockade of Ca^+2^ channels. Interestingly, Ca^+2^-channel blocking is known to be useful as tracheal relaxant in disorders characterized by hyperresponsiveness of respiratory tract [23–25]. 

Moreover, the extract caused relaxation of both phenylephrine (1 *μ*M)- and K^+^ (80 mM)-induced contractions in isolated rabbit aorta preparations in a concentration-dependent manner. Phenylephrine causes an increase in the tone of the vascular smooth muscles by an increase in Ca^+2^ influx via two means, that is, influx of Ca^+2^ via receptor operator channels and through release of intracellular Ca^+2^ [26]. Hence, the observed relaxation of both phenylephrine- and K^+^ (80 mM)-induced contractions may be viewed as nonspecific vasodilator action likely to be mediated through the blockade of Ca^+2^ channels and Ca^+2^ channel blocking agents are being prescribed as vasodilators in the management of congestive heart failure and hypertension [20, 27].

On the basis of above-mentioned, these data contribute to validate the folkloric use of *Prosopis cineraria* for the management of gastrointestinal and respiratory ailments.
